# Imaging Dynamics Beneath Turbid Media via Parallelized Single‐Photon Detection

**DOI:** 10.1002/advs.202201885

**Published:** 2022-06-24

**Authors:** Shiqi Xu, Xi Yang, Wenhui Liu, Joakim Jönsson, Ruobing Qian, Pavan Chandra Konda, Kevin C. Zhou, Lucas Kreiß, Haoqian Wang, Qionghai Dai, Edouard Berrocal, Roarke Horstmeyer

**Affiliations:** ^1^ Department of Biomedical Engineering Duke University Durham NC 27708 USA; ^2^ Department of Automation Tsinghua University Beijing 100084 China; ^3^ Division of Combustion Physics Department of Physics Lund University Lund 22100 Sweden; ^4^ Institute of Medical Biotechnology Friedrich‐Alexander‐University Erlangen‐Nürnberg (FAU) Erlangen 91054 Germany; ^5^ Tsinghua Shenzhen International Graduate School Tsinghua University Shenzhen 518055 China; ^6^ Department of Electrical and Computer Engineering Duke University Durham NC 27708 USA; ^7^ Department of Physics Duke University Durham NC 27708 USA

**Keywords:** deep imaging, dynamic scattering, single‐photon avalanche diode array

## Abstract

Noninvasive optical imaging through dynamic scattering media has numerous important biomedical applications but still remains a challenging task. While standard diffuse imaging methods measure optical absorption or fluorescent emission, it is also well‐established that the temporal correlation of scattered coherent light diffuses through tissue much like optical intensity. Few works to date, however, have aimed to experimentally measure and process such temporal correlation data to demonstrate deep‐tissue video reconstruction of decorrelation dynamics. In this work, a single‐photon avalanche diode array camera is utilized to simultaneously monitor the temporal dynamics of speckle fluctuations at the single‐photon level from 12 different phantom tissue surface locations delivered via a customized fiber bundle array. Then a deep neural network is applied to convert the acquired single‐photon measurements into video of scattering dynamics beneath rapidly decorrelating tissue phantoms. The ability to reconstruct images of transient (0.1–0.4 s) dynamic events occurring up to 8 mm beneath a decorrelating tissue phantom with millimeter‐scale resolution is demonstrated, and it is highlighted how the model can flexibly extend to monitor flow speed within buried phantom vessels.

## Graphical abstract

The work presents a new non‐invasive way to image dynamic events occurring deep within rapidly decorrelating, diffusely scattering media. By combining a novel single‐photon avalanche diode array with a custom‐designed fiber bundle probe and a new data‐driven approach, the work demonstrates the ability to reconstruct images of transient dynamic events beneath decorrelating tissue phantoms.

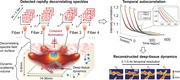

## 1 Introduction

Imaging deep within human tissue is a central challenge in biomedical optics. Over the past several decades, a wide variety of approaches have been developed to address this challenge at various scales. These include confocal^[^ ^1^ ^]^ and nonlinear^[^ ^2^ ^]^ microscopy techniques that can image up to 1 mm deep within tissue, as well as novel wavefront shaping,^[^ ^3^ ^]^ time‐of‐flight diffuse optics,^[^ ^4^, ^5^ ^]^ and photoacoustic techniques^[^ ^6^ ^]^ that can extend imaging depths to centimeter scales at reduced resolution. While there are many experimental demonstrations of imaging through thick scattering material, only a few of these techniques can easily be translated to living tissue specifically, or to dynamic scattering media in general. Dynamic scattering specimens, such as tissue decorrelate^[^ ^7^ ^]^—microscopic movements due to effects like thermal variations and cell migration, for example, cause the optical scattering signature of a particular specimen to change rapidly over time. This rapid movement often presents challenges to effective in vivo deep‐tissue imaging. While prior wavefront shaping methods can overcome such effects to focus within thick tissue at high speeds,^[^ ^8^, ^9^, ^10^ ^]^ significant engineering challenges remain to achieve deep‐tissue imaging in human subjects.^[^ ^11^ ^]^

Instead of attempting to avoid or overcome the effects of decorrelation on imaging measurements, one alternative strategy is to directly measure such dynamic changes within the scattering specimens, and use these changes to aid with image formation. Here, the primary goal is not to form intensity‐based images, as in absorption or fluorescence microscopy, but to create a spatial map of fluctuation. This is typically achieved by measuring the temporal dynamics (e.g., temporal variance or correlation) of scattered radiation. Several important biological phenomena cause such temporal variation of an optical field, ranging from blood flow to neuronal firing events.^[^ ^12^, ^13^, ^14^, ^15^ ^]^ Optical coherence tomography angiography,^[^ ^16^ ^]^ laser speckle contrast imaging,^[^ ^13^ ^]^ as well as photoacoustic Doppler microscope^[^ ^17^ ^]^ have been developed to image such dynamics close to the tissue surface. However, to detect an optical signal that has traveled deep inside living tissue, which increasingly attenuates and decorrelates the optical field, one typically needs to eventually rely on fast single‐photon‐sensitive detection techniques that record optical fluctuations at approximately mega‐hertz rates.

One established technique to detect dynamic scattering multiple centimeters within deep tissue is termed diffuse correlation spectroscopy (DCS),^[^ ^18^ ^]^ which records coherent light fluctuations. When coherent light enters a turbid medium, it randomly scatters and produces speckle. Movements within the tissue volume (e.g., cellular movement or blood flow) occur at different spatial locations and interact with the scattered optical field. By measuring temporal fluctuations of the scattered light at the tissue surface, it is possible to estimate a spatiotemporal map of decorrelating events. While such methods are widely used to assess blood flow variations across finite tissue areas as deep as beneath the adult skull,^[^ ^19^ ^]^ there has been limited work to date to rapidly form spatially resolved images and video of dynamic events beneath turbid media,^[^ ^18^ ^]^ despite early work demonstrating that the temporal correlation of light transports through tissue follows a well‐known diffusion process.^[^ ^20^ ^]^ Three main challenges have prevented imaging of deep‐tissue dynamics: 1) a low signal‐to‐noise (SNR) due to a limited number of available photons at requisite measurement rates, 2) a limited number of detectors to collect light from different locations across the scatterer surface, and 3) a challenging ill‐posed inverse problem to map acquired data to accurate imagery.

To solve the first two challenges listed above, this work uses a single‐photon avalanche diode (SPAD) array to simultaneously measure speckle field fluctuations across the tissue surface at the requisite sampling rates (≈μs) and single‐photon sensitivities needed for deep detection.^[^ ^21^ ^]^ Recently developed SPAD arrays, based on standard complementary metal‐oxide–semiconductor(CMOS) fabrication technology, can integrate up to a million SPAD pixels onto a small chip.^[^ ^22^, ^23^ ^]^ This led to new imaging applications in fluorescence lifetime imaging,^[^ ^24^ ^]^ scanning microscopy,^[^ ^25^ ^]^ confocal fluorescence fluctuation spectroscopy,^[^ ^26^ ^]^ Fourier ptychography,^[^ ^27^ ^]^ as well as computer vision tasks, such as depth profile estimation,^[^ ^22^, ^28^ ^]^ seeing around corners^[^ ^29^ ^]^ and through scattering slabs.^[^ ^4^, ^30^ ^]^ Most prior DCS measurement systems relied on fast single‐pixel single‐photon detectors (including single‐pixel SPAD and photomultiplier tubes) for optical measurement.^[^ ^18^ ^]^ Single‐pixel strategies for DCS‐based image formation have several fundamental limitations. While several works demonstrated DCS‐based imaging of temporal correlations in the past,^[^ ^14^, ^20^, ^31^, ^32^, ^33^ ^]^ none simultaneously acquired DCS signal from multiple tissue surface areas, as required for rapid image formation (e.g. to avoid effects of subject movement). Instead, these prior works mechanically scanned the specimen, or illumination and detection locations in a step‐and‐repeat fashion to measure speckle from different surface locations on a single detector. Furthermore, as only one or a few speckle modes can be sampled by a single detector while still maintaining suitable contrast, a long (seconds or more) measurement sequence is typically required to obtain a suitable signal‐to‐noise ratio for each measured temporal correlation curve (i.e., each surface location). This limited correlation measurement rate is quite detrimental—it precludes observation of dynamic variations of the subject pulse signal, for example, which can vary at sub‐hertz rates. Recent work has demonstrated how parallelized speckle detection across many optical sensor pixels^[^ ^34^, ^35^, ^36^, ^37^, ^38^ ^]^ can lead to significantly faster correlation sampling rates. We build upon these insights to create a new system capable of recording spatially resolved videos of temporal decorrelation without any moving parts.

**Figure 1:**
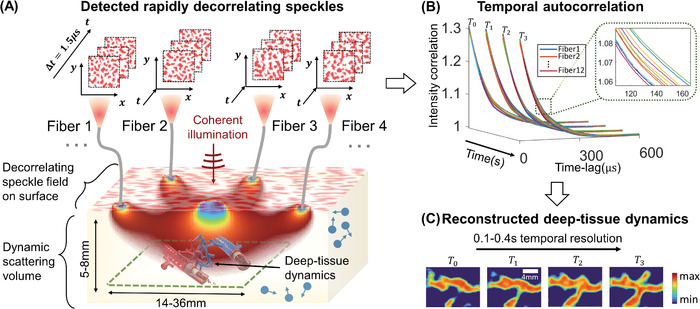
Flow diagram of proposed method for imaging temporal decorrelation dynamics. A) Illustration of parallelized diffuse correlation imaging (PaDI) measurement strategy. Scattered coherent light from source to multiple detector fibers travels through decorrelating scattering media along unique banana‐shaped paths. Fully developed speckle on the tissue surface rapidly fluctuates as a function of deep‐tissue movement. Green dashed box marks deep‐tissue dynamics areas of interest for imaging. B) Computed autocorrelation curves from time‐resolved measurements of surface speckle at different tissue surface locations. C) Autocorrelation variations caused by deep‐tissue dynamics are computationally mapped into spatially resolved images of transient dynamics.

The third challenge noted above relates to the computational formation of dynamic images from limited measurement locations across the scatterer surface, typically formulated as an ill‐posed inverse diffusion problem. While model‐based solvers have demonstrated effective dynamics imaging in prior work,^[^ ^14^, ^20^, ^31^, ^32^, ^33^ ^]^ simple scattering geometries were typically assumed (e.g., infinite and semi‐infinite geometries). To alleviate model‐based reconstruction issues, one can adopt a data‐driven image reconstruction approach. Typically formed via training of a nonlinear estimator with large amount of labeled data, neural network‐based models have been used in the past to image static amplitude or phase objects through and within scattering medium using both all‐optics^[^ ^39^, ^40^, ^41^, ^42^, ^43^, ^44^ ^]^ and photoacoustic methods.^[^ ^45^ ^]^ Inspired by such recent progress, we have developed a system and data post‐processing pipeline, termed parallelized diffuse correlation imaging (PaDI), that addresses the above challenges to form images and video of transient dynamics events beneath multiple millimeters of decorrelating turbid media. Our new optical probe can image within a 140 mm^2^ field‐of‐view at 5–8 mm depths beneath a decorrelating liquid tissue phantom (μ_a_ = 0.01 mm^−1^, ${\mu^{\prime }}_{s}=0.7$ mm^−1^, Brownian coefficient *D* = 1.5 × 10^6^ mm^2^, for example—although many of these parameters can be flexibly adjusted) without any moving parts at multi‐hertz video frame rate. **Figure** **1** presents an overview of the proposed method.

## 2 Results and Discussion

### 2.1 PaDI

**Figure 2:**
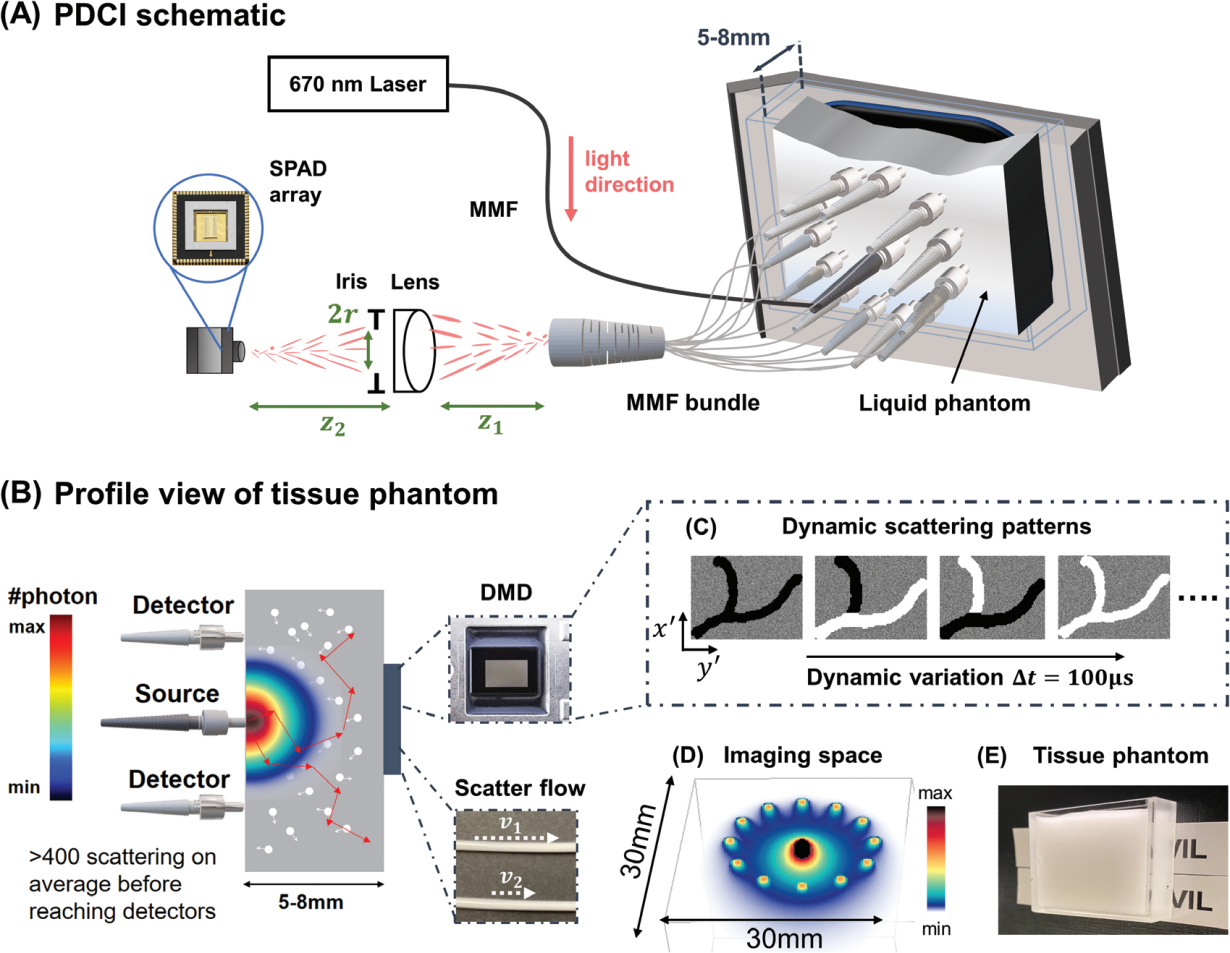
A) Schematic of PaDI system for imaging decorrelation. Back‐scattered coherent light from single input port is collected by 12 multimode fibers (MMF) at tissue phantom surface and guided to SPAD array camera. B) Profile view of the tissue phantom imaging experiment. Digital micro‐mirror device (DMD) and vessel phantom serve as source of temporal dynamics and is hidden beneath phantom by placing it immediately adjacent (separated by coverglass). All sources and detectors are placed on the same side of phantom. Colormap provides qualitative photon distribution map, where quantitative plot of sub‐surface photon distribution is in Figure S1B, Supporting Information. C) A set of DMD patterns that can be used to generate spatiotemporal varying dynamics. (D) Simulation of photon‐sensitive region of our 12‐fiber system. (E) A picture of the tissue phantom we use in experiments.

The phantom design and imaging setup is outlined in **Figure** **2**. To assess the performance of our PaDI system, we turn to an easily reconfigurable nonbiological liquid phantom setup that offers the ability to flexibly generate unique image targets with known spatial and temporal properties. To mimic decorrelation rates and scattering properties of human tissue, we utilized a liquid phantom filled with 1 µm‐diameter polystyrene microspheres (4.55 × 10^6^# mm^−3^) solution enclosed in a custom‐designed thin‐walled cuvette as rapidly decorrelating turbid volume to occlude the target of interest. The target exhibits a reduced scattering coefficient of 0.7mm^−1^ as computed by the Lorenz–Mie method, and an experimentally measured absorption coefficient of 0.01mm^−1^. Also, based on fitting using a Monte Carlo method,^[^ ^46^ ^]^ the medium exhibits an estimated Brownian motion diffuse constant of 1.5 × 10^6^mm^2^, which is close to the diffusion coefficient measured in model organisms.^[^ ^47^ ^]^ Section S3, Supporting Information details how these values are estimated. To generate expected temporal fluctuation variations within living tissue caused, for example, by blood flow, we placed a digital micro‐mirror device (DMD) immediately behind this tissue phantom, with which we computationally created spatiotemporally varying patterns at kilohertz rates.^[^ ^35^ ^]^ Further, for the second generalizability study discussed in Section 2.3, we also place two plastic tubes containing flowing scattering liquid with the same optical properties as the background volume. The movement of the liquid inside the tube is controlled with two syringe pumps (New Era, US1010).

Our light source is a 670 nm diode‐pumped solid‐state (DPSS) laser (MSL‐FN‐671, Opto Engine LLC, USA) with a coherence length ⩾10 m, which we attenuated to 200 mW to match standard ANSI safety limits for illuminating tissue with visible light.^[^ ^48^ ^]^ We guided this light to the liquid phantom surface using a 50 µm, 0.22 numerical aperture (NA) multi‐mode fiber (MMF). Before the MMF, we ensured that the DPSS laser output was effectively a single transverse mode with a fiber coupler, such that either an MMF or a single‐mode fiber (SMF) could serve as the source waveguide,^[^ ^35^, ^36^ ^]^ with MMF being a generally less expensive option. After entering the liquid phantom, the light randomly scatters and decorrelates, and a small fraction of which reaches the DMD placed immediately behind the turbid medium. The side of the phantom cuvette facing the DMD is made of microscope slide coverglass. Each square DMD pixel has 13.7 × 13.7 µm^2^ area. With 768 × 1024 pixels, the entire DMD panel has a screen size of 10.4 × 13.9mm^2^. We chose to use a DMD to generate the spatiotemporal dynamic scattering patterns first because it is easily configurable: light reaching the quickly flipping pixels decorrelates faster than light that does not, and these pixels are digitally addressable and thus can be changed both spatially and temporally without moving the setup. Second, because it can meet requisite dynamic variation speeds (we run the DMD between 5–10 kHz), which we have selected to correlate with the response of blood flow at tested depths (5–8 mm).^[^ ^35^ ^]^ As the reflected multi‐scattered light penetrates on average about $\frac{1}{2}-\frac{2}{3}$ times the source‐detector distance (ρ) deep into the phantom tissue,^[^ ^49^ ^]^ we place 12 multi‐speckle detection fibers circularly around the source in the center with ρ = 9.0mm. Each multi‐speckle detection fiber is a MMF with a 250 µm core diameter and 0.5 NA. Quantitative plots of an *x*–*z* cross section of the most probable scattered and collected photon trajectories, as well as the expected number of photons per speckle per sampling period, are provided in Figure S1B, Supporting Information. We use a modern Monte Carlo simulator called “multi‐scattering”^[^ ^50^ ^]^ that models anisotropy from spherical scattering centers using a Lorenz–Mie based scattering phase functions. The model has recently been rigorously validated against experimental results as shown in refs. [51, 52] and can obtain 3D representations of photon paths within the simulated scattering medium. Such results are shown in Figure 2C for the experimental configuration presented in this article, where 12 optical fibers are used for collecting photons, which is the imaging space of our PaDI system. Visualizations of 3D trajectories for detected photon using different numbers of fibers are also provided in Figure S2B, Supporting Information. Away from the tissue surface, the distal ends of the 12 MMFs are bundled together and imaged onto the SPAD array (PF32, Photon Force, UK) with a magnification $M=\frac{z_{2}}{z_{1}}$ using a single lens with an iris diaphragm placed directly adjacent to the lens. As labeled in Figure 2B, *r*, *z*_1_, and *z*_2_ are the radius of the iris diaphragm, the distance between fiber bundle exist and lens, and the distance between lens and SPAD array sensor plane, respectively. To form an image of the fiber bundle on the camera, *z*_1_ and *z*_2_ satisfy the thin lens equation. In practice, $\frac{r}{z_{1}}$ is much smaller than the fiber NA that we choose, which determines the NA of the overall speckle imaging system. As illustrated in Figure S4B, Supporting Information, the 32 × 32 SPAD array has an overall size of 1.6 × 1.6 mm^2^ with a pixel pitch of *w*_p_ = 50 µm and an active area that is ϕ = 6.95 µm in diameter. As the magnification is fixed for imaging the light exiting the fiber bundle onto the whole camera, we tune the radius of the iris diaphragm to alter the average speckle size, such that ≈1 speckle on average is mapped onto each SPAD pixel active area; that is, we want the speckle size on the sensor plane to match ϕ. Given that the collected light experiences ≈440 scattering events on average (see Figure S1C, Supporting Information), the emerging light at the tissue surface is a fully developed speckle pattern with an average speckle size of $\frac{\lambda }{2}$ ^[^ ^53^ ^]^ and uniformly distributed phase.^[^ ^54^ ^]^ Hence, setting *M*λ/2NA = ϕ gives the desired iris radius $r=\frac{\lambda M}{2\phi z_{1}}$.

### 2.2 Supervised Learning for Image Reconstruction

For our first demonstration of PaDI, we use an artificial neural network to reconstruct images and video of deep temporal dynamics from measured surface speckle intensity autocorrelation curves. As detailed in “Parallelized Diffuse Correlation Imaging” and “Data Acquisition and Preprocessing” sections, we collect speckles from 12 distinct surface positions using multimode fibers (MMF), and estimate the intensity autocorrelation for each location. Each intensity autocorrelation curve has 400 sampled time‐lags (1.5 µs sampling rate). There are 12 such curves, each computed from the associated SPAD pixels that measure scattered light from the PaDI probe's 12 fiber detectors. A new set of such 12 curves is produced every frame integration time *T*_int_ (variable between 0.1 and 0.4 s). Combining and vectorizing our system's 12 autocorrelation curves gives the neural network input, $x\in R^{4800}$. The output of the neural network is an image $x\in R^{48\times 64}$, with an image pixel size of 220 × 220 µm^2^. This pixel size is a tunable parameter in our reconstruction model, which we select as smaller than the expected achievable resolution^[^ ^18^, ^35^ ^]^).

**Figure 3:**
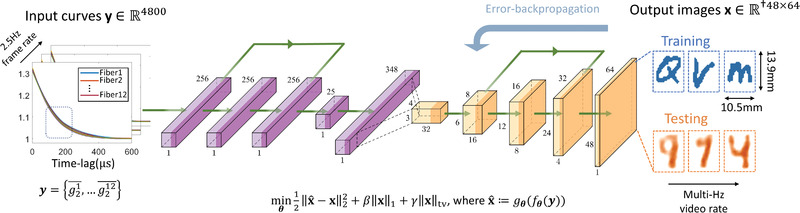
Proposed artificial neural network architecture for PaDI reconstruction, which takes a set of 12 computed intensity auto‐correlation curves as input. The network first encodes the high‐dimension measurement into a low‐dimension manifold through a stack of fully‐concerted layers, and decodes the embedding into a spatial reconstructions of the dynamics hidden underneath decorrelating phantom tissue, using convolutional layers. Bent green arrows are skip connections.

**Figure** **3** depicts our image reconstruction network. While prior works^[^ ^40^, ^41^, ^42^, ^43^, ^44^, ^45^ ^]^ have used image‐to‐image translation networks to form images of fixed objects through scattering material, our reconstruction task here is quite different from these alternative networks and thus required us to develop a tailored network architecture. First, the format of our network input is unique (multiple autocorrelations created from noninvasive measurement of second‐order temporal statistics of scattered light). Second, the contrast mechanism of our network output is also different—a spatial map of dynamic variation described by speed of change per pixel. Our network mapping problem (multi‐autocorrelation inputs into spatial maps of temporal dynamics) is thus in some ways similar to domain transform problems. Therefore, our employed network design is most similar to that introduced by Zhu et al.^[^ ^55^ ^]^ Overall, the network is composed of an encoder *f*_θ_(·) to compress the input into a low‐dimensional manifold, and a decoder *g*_θ_(·) to retrieve the spatial map of temporal dynamics from the embedding. The encoder is composed of three fully‐connected layers, with skip connections to allow the error to propagate more easily. All fully‐connected layers uses leaky‐ReLU activation functions with a slope of 0.1, and the first three fully‐connected layers have a dropout rate of 0.05. After the inputs are embedded into a low‐dimensional manifold, the decoder maps the embedding into the 2D reconstruction of dynamics using five transposed convolution layers with stride 2 and padding 1. The network is updated to solve the following problem

(1) $$\min_{\theta }\sum_{i=1}^{M}\left(D\left(x^{i},{\hat{x}}^{i}\right)+R\left({\hat{x}}^{i}\right)\right)$$

where ${\hat{x}}^{i}:=g_{\theta }\left(f_{\theta }\left(y^{i}\right)\right)$ is the output of the network from *i*th set of measurements $y_{i}$, and *M* is the total number of training pairs.

(2) $$D\left({\hat{x}}^{i},x^{i}\right)=\frac{1}{2}∥{\hat{x}}^{i}-x^{i}{∥}_{2}^{2}$$

is the data‐fidelity term that train the network to find prediction that matches the ground truth, and

(3) $$R\left({\hat{x}}^{i}\right)=\lambda ∥{\hat{x}}^{i}{∥}_{1}+\zeta \mathrm{TV}\left({\hat{x}}^{i}\right)$$

The ℓ_1_ norm is used to promote sparsity of the reconstruction, and TV(·) is the isotropic total variation penalty that makes the reconstruction piecewise constant. These regularizations have been successfully applied to improve diffuse optics imaging reconstructions.^[^ ^56^, ^57^ ^]^ λ and ζ are hyperparameters empirically chosen to be 0.02 and 0.1, respectively, to balance the data fidelity and image prior knowledge. As we have a small dataset for training, we do not divide the data further to create a validation dataset. Instead, we apply early stop to avoid overfitting. The networks for all tasks used Xavier initialization^[^ ^58^ ^]^ and trained for 2000 epochs using the Adam optimizer^[^ ^59^ ^]^ with a 8 × 10^−4^ learning rate and 256 batch size.

### 2.3 Experimental Validation with Digital and Bio‐Inspired Phantoms

We validated our learning‐based image reconstruction method with four unique experiments that each utilized a unique training data set. First, since detecting deep‐tissue blood flow is a primary aim of PaDI system development, we studied the ability of our network to image vessel‐like structures using 1428 vasculature patterns extracted from biomedical image data^[^ ^60^ ^]^ (1190 for training, 238 for testing). Image data was rescaled to an appropriate size (10.4 × 13.9 mm^2^) and displayed as a dynamic pattern with a 5 kHz variation rate. By comparing PaDI and standard inverse diffusion model‐based reconstructions (as detailed in Section S3, Supporting Information), we highlight significant improvement. Second, we tested the generalizability of PaDI by training the network with objects drawn from one type of dataset, and testing the network with objects drawn from a second distinct dataset type (i.e., from a different distribution). For this generalizability experiment, we trained with 1280 hand‐written letters from the EMNIST dataset and assessed reconstruction accuracy used 128 digits from the MNIST dataset during algorithm testing. Third, we explored the potential of our method to jointly image both temporal and spatially varying dynamic potentials by using PaDI to image objects of different sizes and unique fluctuation rates (5 and 10 kHz). Finally, we further tested system generalizability by acquiring PaDI data from a completely unique phantom tissue arrangement, containing 3 mm diameter phantom vessels buried 5 mm beneath scattering material, through which we flowed liquid at variable speeds. We then applied a DMD‐trained image formation model, trained with 1046 patterns containing two tube‐shape objected demonstrated in Figure 6A, to spatio‐temporally resolve buried capillary flow dynamics, highlighting the flexibility of both the imaging hardware and post‐processing software.

**Figure 4:**
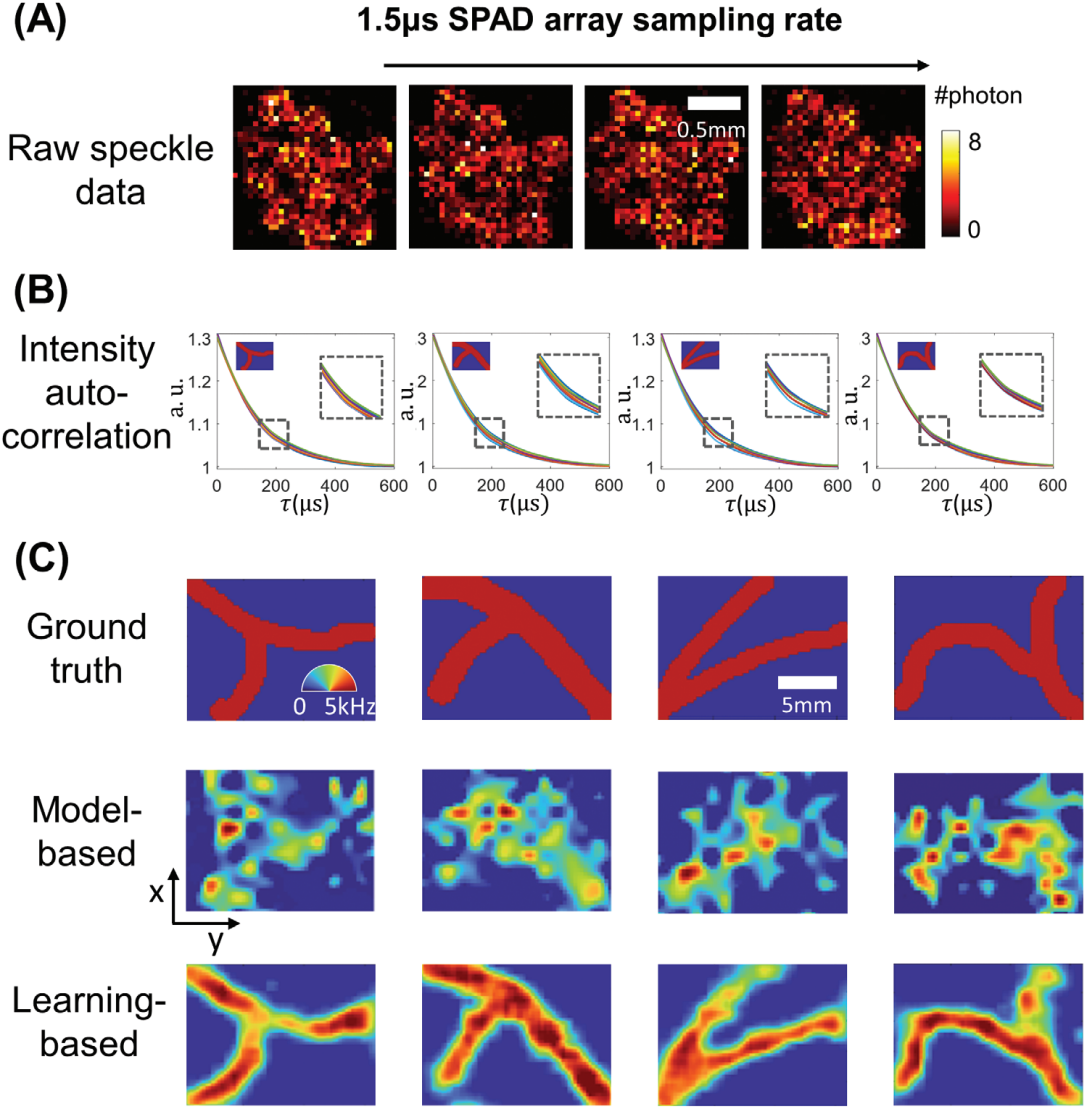
PaDI measurements and reconstructions of phantom vasculature patterns located 5 mm beneath a tissue‐like decorrelating turbid volume. A) Recorded raw SPAD array speckle intensity (colorbar: photons detected per pixel). B) Processed intensity auto‐correlations using *T*_int_ = 0.4 s where *x*‐axis is time‐lag τ. Each plot labeled with ground truth of dynamic scattering image on the top‐left, with zoom‐ins showing curve regions most sensitive to spatially varying decorrelation. C) Ground truth dynamic scattering object 5 mm beneath tissue phantom with PaDI reconstructions using a model‐based method (for comparison) and proposed learning‐based method. All figures in (C) share same color wheel (dynamic scatter fluctuation rate), scale bar, and *x*–*y* coordinates

**Figure 5:**
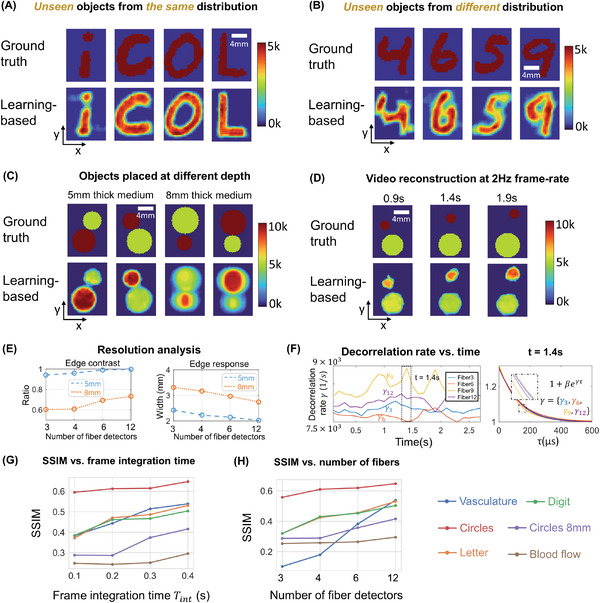
PaDI reconstructions of spatiotemporal dynamics for various patterns and decorrelation speeds hidden beneath 5–8 mm thick turbid volume. A) Reconstructions of letter‐shaped dynamic scatter patterns hidden underneath 5 mm turbid volume, sampled from a distribution that matches training data distribution. B) Reconstructions of digit‐shaped dynamic scatter patterns hidden underneath 5 mm turbid volume, drawn from a different distribution as compared to training data distribution. C) Reconstructions of objects at varying dynamic scattering rate hidden beneath 5 and 8 mm‐thick turbid volume, along with E) resolution analyses for different depths using different number of fiber detectors. D) A few reconstruction frames from a video taken over 3 s. F) plots four of decorrelation rates change in time. A set of autocorrelation curves from these four‐fiber detection at 1.4 s is presented on the right. G) Plots of average SSIM between ground‐truth and reconstructed speed maps as a function of frame integration time *T*_int_ for various tested datasets. H) Plots of average SSIM between ground‐truth and reconstructed speed maps as a function of number of detection fibers used for image formation. (G) and (H) share the same legend listed at the bottom of the figure. Imaging datasets are described in the Section 2.3.

**Figure** **4**A shows a few representative raw SPAD array measurements (1.5 µs exposure time). 12 circular spots in the raw frame are roughly discernible. Each spot contains photon count statistics of scattered light collected from one of 12 different locations on the tissue phantom surface and delivered to the array via MMF. Figure 4B plots the intensity autocorrelation curves for each of the 12 unique SPAD array regions (i.e., each unique location on the tissue phantom surface). These curves are averages computed over space (all SPAD measurements per fiber) and time (a frame integration time here of 0.4 s). The dynamic scattering patterns used to generate each set of auto‐correlation curves are labeled on the upper right corner of each plot, and the regions most sensitive to the perturbations are enlarged.

The first row of Figure 4C displays several examples of dynamic patterns from the vasculature dataset produced in our phantom setup beneath 5 mm of turbid decorrelating media. The second and third rows show PaDI reconstructions for these patterns using our proposed learning‐based method and a regularized model‐based reconstruction method, for comparison. Details regarding the model‐based reconstruction method can be found in Section S3, SUpporting Information. Due to the ill‐posed nature of the inverse problem and model‐experiment mismatch, model‐based reconstruction results are less spatially informative compared to our proposed learning‐based method, even when strong structural image priors are used. We observe some marginal artifacts in reconstructions using the proposed learning‐based method, where the reconstructed edge values are typically lower than the ground‐truth, as the high frequency on the edge is harder to reconstruct. While **Figure** **5**A shows the dynamic scattering potential reconstructions for unseen objects drawn from a distribution that matches the training dataset, Figure 5B shows dynamic scattering reconstructions for unseen objects drawn from a different distribution as compared to the training dataset. These results suggest that the trained network has the generalizability to predict unseen dynamic scattering objects that have limited correlation with expectation. At the same time, we also observe that the reconstructions for the objects drawn from a different distribution are less sharp than reconstructions for objects drawn from the same distribution as the training set, even though the average structural similarity index measure (SSIM)^[^ ^61^ ^]^ values between the two testing datasets are comparable, as shown in Figure 5G–H.

**Figure 6:**
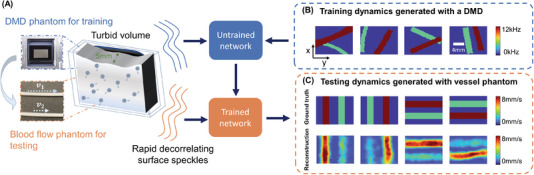
A) Illustration of deep tissue phantom capillary flow experiment. PaDI network is first trained on synthetic data generated by DMD phantom, then applied to reconstruct images from separate capillary flow phantom setup. B) Examples of dynamic scattering patterns used for training, generated at up to 12 kHz on DMD phantom. C) Representative images reconstructed with proposed learning‐based method, along with ground truth. Dynamics are generated with two capillary tubes buried beneath a 5 mm scattering volume exhibiting variable‐speed liquid flow.

Next, we tested the ability of PaDI to resolve decorrelation speed maps that vary as a function of space and at different phantom tissue depths. PaDI reconstructions for two variable‐speed perturbations under both 5 and 8 mm of turbid medium are in Figure 5C. In this experiment, 1280 and 108 patterns of variable speed and shape were utilized for training and testing, respectively. First, we observe that PaDI can spatially resolve features while still maintaining an accurate measure of unique decorrelation speeds. When structures with different decorrelation speeds begin to spatially overlap, the associated reconstructed speed values close to the overlap boundary are either lifted or lowered toward that of the neighboring structure. This is expected, as the detected light travelling through the “banana‐shaped” light path contains information integrated over a finite‐sized sensitivity region that will effectively limit the spatial resolution of the associated speed map reconstruction. Moreover, we also observed that PaDI reconstructions of dynamics hidden beneath a thicker 8 mm scattering medium are less accurate than those for dynamics beneath a 5 mm scattering medium. A resolution analysis based on the contrast in the edge regions of the circles, and the width of 10–90% edge response is also provided in Figure 5E. Speckle fluctuations sampled by our current configuration on the phantom tissue surface are less sensitive to decorrelation events occurring within deeper region. Creating a PaDI probe with larger source‐detector separations can help address this challenge, as detailed in the Section 2.4. Further, we collect continuous data for 3 s, where the dynamic patterns hidden underneath present for 0.3 s, and change every 0.5 s. We show reconstructions of a few frames at Figure 5D. Figure 5F plots four of decorrelation rates change in time. The decorrelation rates are extracted by fitting each autocorrelation curves (using a Levenberg–Marquardt algorithm) with 1 + β*e* ^γτ^, where τ is delay‐time and γ is the decorrelation rates. These autocorrelation curves are used to generate reconstructions in Figure 5D. 3 s continuous measurements are taken, and the curves are estimated using 0.3 s integration window and 66.7% overlap between sliding windows. A set of autocorrelation curves from these four‐fiber detection at 1.4 s is presented on the right. We additionally conducted an experiment to study how our model, trained with data generated on our digital phantom, can reconstruct images of the dynamic scattering introduced by more biologically realistic contrast mechanisms. Noninvasive imaging of deep blood flow dynamics, such as hemodynamics within the human brain, is an important application for diffuse optical correlation‐based measurements. Accordingly, we modeled deep hemodynamic flow by placing two capillary tubes (3 mm diameter) directly beneath a dynamic scattering volume (same optical properties: μ_a_ = 0.01 mm^−1^, ${\mu^{\prime }}_{s}=0.7\;{\mathrm{mm}}^{-1}$) flowing at two different speeds (2.7 and 8.0 mm s^−1^) via syringe pump injection. After training an image formation model with PaDI data captured on our DMD‐based phantom (630 maps of randomly oriented tube‐like objects varying at 4–12 kHz, see **Figure** **6**A), we acquired PaDI data from this unique capillary flow phantom and applied the DMD phantom‐trained model to produce images as shown in Figure 6B. Here, we observe reconstructed image measurements of relative flow speed with spatial and temporal structures that match ground truth, pointing toward a system that can potentially image dynamic scattering beneath tissue in vivo using learning‐based reconstruction methods trained with more easily accessible synthetic data.

Finally, we assessed experimental PaDI performance as a function of detection speed and number of spatial measurement points using the SSIM metric,^[^ ^61^ ^]^ as more than one contrast mechanism (DMD, fluid dynamics) used in different experiments. Figure 5G,H plot average SSIM as a function of frame integration time *T*_int_ and as a function of number of surface detectors *P* for all datasets above. Figure 5G's data used all 12 unique phantom surface locations for its reconstructions. 5(H)'s data used a 0.4 s frame integration time. From these plots, it is clear that a longer frame integration time improves reconstruction performance, at the expense of a proportionally decreased PaDI frame rate. In addition, collecting speckle dynamics from more surface locations improves the reconstruction results, as expected. This is not only because the imaging (photon‐sensitive) region of the 12‐fiber system is larger than that using fewer fibers, but also because the overlap between banana‐shaped photon paths from adjacent fiber detectors (e.g., see Figure S2, Supporting Information) provides redundant data that is beneficial to accurate image formation.

### 2.4 Discussion

In summary, we have developed a new parallelized speckle sensing method that can spatially resolve maps of decorrelation dynamics that occur beneath multiple millimeters of tissue‐like scattering media. Our approach utilizes diffuse correlation principles to sample speckle fluctuations from different locations along a scattering medium's surface at high speed. Unlike prior work, our system records all such measurements in parallel to reconstruct transient speed maps at multi‐hertz video frame rate, and uses a novel machine learning approach for this reconstruction task that outperforms standard model‐based solvers.

While we demonstrated that PaDI can rapidly image dynamic events occurring under a decorrelating tissue phantom, several potential improvements can be made to ensure effective translation into in vivo use. First, as shown in the raw speckle data from the SPAD array, the fiber bundle we use was not optimized to maximize the speckle detection efficiency—our fiber bundle array did not map surface speckle to all SPADs within the array. Future work will endeavor to utilize a custom‐designed fiber bundle that provides better array coverage. We note that detection efficiency was further reduced in our phantom setup by the cover glass surfaces on both sides of the cuvette holding the liquid tissue phantom, both via reflection and by enforcing a finite standoff distance from the phantom for the fiber probe, which decreased light collection efficiency. This can be resolved in the future using a more suitable material,^[^ ^62^ ^]^ which we expect to further improve the sensitivity of our PaDI system. In addition, we used a DMD in this work to generate simulated deep‐tissue dynamics because it provided an easily reconfigurable means to assess performance for a variety of decorrelating structures. The use of a DMD restricted the total lateral dimension of the phantom tissue and hidden structure that we were able to probe, which additionally prevented us from being able to investigate larger source‐detector separations that are well‐known to improve detection accuracy for deeper dynamics. Based upon the findings in this work, a tissue phantom with embedded vessel phantoms containing flowing liquid can be designed to provide additional verification of PaDI imaging performance at greater depths.^[^ ^63^ ^]^ Recently developed time‐of‐flight^[^ ^64^, ^65^ ^]^ methods also enhance signal from greater depths and can be considered as additional avenues through which PaDI can be improved.

In the future, we also plan to study how our system can jointly image blood flow at variable oxygenation levels. By adding an isosbestic wavelength to the current system, we can potentially spatially resolve blood flow speed as a function of oxygen level. On the computational side, one of the problems of using classic supervised deep learning methods as a maximum likelihood estimator is reconstruction reliability concern. One expensive solution is to expand the training set to include large amount of objects. In this data‐rich scenario, meta‐learning approaches can also be considered, where part of the network weights are allowed to be changed depending on different imaging setups.^[^ ^66^ ^]^ In a resource limited situation, however, an alternative strategy might assess the reliability by predicting the uncertainty along with the reconstruction using approximate deep Bayesian inference.^[^ ^67^ ^]^ These additional investigations will aid with the eventual translation of PaDI into a practical and reliable tool for recording video of deep‐tissue blood flow in in vivo subjects in the future.

## 3 Experimental Section

### Data Acquisition and Preprocessing

The SPAD array's 1024 (32 × 32) independent single SPADs were used to count photons arriving at each pixel with a frame rate of 666 kHz and a bit depth of 4. This is equivalent to an exposure time of *T*_s_ = 1.5 µs. To extract the temporal statistics from measurements of randomly fluctuating surface speckle at 666 kHz, then a temporal autocorrelation was computed on a per‐SPAD basis. Although it was noted that there were a number of strategies available to compute such temporal statistics across a SPAD array (e.g., joint processing across pixels, examining higher‐order statistics, or more advanced autocorrelation inference methods^[^ ^68^, ^69^ ^]^), the per‐pixel method was selected here as it was well‐established.^[^ ^34^, ^35^, ^36^ ^]^ The temporal autocorrelations was computed across “frame integration time” of typically *T*_int_ = 0.4 s, which yielded N = *T*_int_/*T*_s_ frames per autocorrelation measurement. Rather than using a physical correlator module, the time‐resolved photon stream was recorded as a 1024 × *N* array and compute the autocorrelations in software, where typically *N* = 266 k. The effect of using a shorter *T*_int_ and fewer SPADs per measurement were also explored and the results were compared in Figure 5G,H.

As illustrated in Figure S4, Supporting Information, the normalized temporal intensity autocorrelation^[^ ^18^ ^]^ of each pixel were computed as

(4) $$g_{2}^{p,q}\left(\tau \right)=\frac{{\left\langle I^{p,q}\left(t\right)I^{p,q}\left(t+\tau \right)\right\rangle }_{T_{\mathrm{int}}}}{{\left\langle I^{p,q}\left(t\right)\right\rangle }_{T_{\mathrm{int}}}^{2}}$$

where *I* ^*p*, *q*^(*t*) is the photon count detected by the *q*th SPAD for *p*th fiber at time *t*; τ is time‐lag (or delay or correlation time), and ${\left\langle \cdot \right\rangle }_{T_{int}}$ denotes time average estimated by integrating over *T*_int_. After calculating $g_{2}^{p,q}\left(\tau \right)$ for each single SPAD, an average, noise‐reduced curve was ten obtained by averaging curves that were produced by the *Q*_p_ unique SPADs that detected light emitted by the same MMF detection fiber

(5) $${\bar{g}}_{2}^{p}\left(\tau \right)=\frac{1}{Q_{p}}\sum_{q=1}^{Q_{p}}g_{2}^{p,q}$$

for the *p*th MMF fiber, where a total of 12 MMF were used. A straightforward calibration procedure allowed to identify the *Q*_p_ SPADs within the array that received light from the *p*th MMF, which was saved as a look‐up table. Next, the $g_{2}^{p}\left(\tau \right)$ was compiled from each fiber into a set of 12 average intensity autocorrelation curves per frame, with the aim of reconstructing the spatiotemporal scattering structure hidden beneath the decorrelating phantom. An example set of intensity autocorrelation curves is in Figure 1B. The maximum lag or delay time τ_max_ was selected at 600 µs, as the values of the intensity autocorrelation started approaching 1 asymptotically.

### Statistical Analysis

In addition to the quantitative analysis reported in Figure 5, here, the computed decorrelation rates γ of the autocorrelation curves for the phantom itself, and when DMD is flickering, was also reported. To estimate decorrelation rates for the phantom when no perturbation occurs, 10 sets of data were captured and computed within 1 h. Each set contained measurements from 12 fibers. From those parallelized speckle sensing data, an average $\bar{\gamma }=8.384\times 10^{3}$s^−1^ was estimated, with a standard deviation σ_γ_ = 0.518 × 10^3^ s^−1^. The average and standard deviation of decorrelation rates for the *Letter*, *Digit*, *Circles* dataset described in Section 2.3 are 8.476 ± 0.396 × 10^3^, 8.480 ± 0.460 × 10^3^, and 8.515 ± 0.435 × 10^3^ s^−1^, respectively. Again, the curves were fitted using a Levenberg–Marquardt algorithm. While this suggests light decorrelated faster on average when the DMD was flicking, a single number was not used to represent how fast the light decorrelated. Instead, all 12 decorrelation curves were used per event for image reconstructions, as described in Section 2.2.

## Conflict of Interest

S.X. and R.H. have submitted a patent application for this work, assigned to Duke University.

## Authors Contribution

S.X., X.Y., W.L.R.Q., and P.C.K. constructed the hardware setup. S.X., W.L., and J. J. designed the software. S.X, K.Z, L.K, E.B., and R.H. wrote the manuscript. H.W., Q.D., E.B., and R.H. supervised the project.

## Supporting information

Supporting Information

## Data Availability

The data that support the findings of this study are available from the corresponding author upon reasonable request.
