# Supplementary material for: Imaging Dynamics Beneath Turbid Media via Parallelized Single‐Photon Detection
Source: Adv Sci (Weinh). 2022 Jun 24;9(24):2201885. doi: 10.1002/advs.202201885 (PMC9404405; doi:10.1002/advs.202201885)
Supplement: Supplementary file 1 — Supporting Information [file ADVS-9-2201885-s001.pdf]

ADVANCED SCIENCE
Open Access

Supporting Information

for *Adv. Sci.*, DOI 10.1002/advs.202201885

Imaging Dynamics Beneath Turbid Media via Parallelized Single-Photon Detection

*Shiqi Xu, Xi Yang, Wenhui Liu, Joakim Jönsson, Ruobing Qian, Pavan Chandra Konda, Kevin C. Zhou, Lucas Kreiß, Haoqian Wang, Qionghai Dai, Edouard Berrocal and Roarke Horstmeyer**

Imaging dynamics beneath turbid media via parallelized single-photon detection: supplementary document

S1 Photon sensitive path and surface fluence analysis

In this section, we present the details of a simulation to studied the photon sensitive path, detected photon number, and scattering distributions of the tissue-like scattering volume used in our experiment. These values and photon-sensitive regions are mentioned repeatedly in the introduction and method sections to motivate the need of a multi-detector, parallelized speckle imaging system and provide valuable insight for our system design. The study uses a recently developed Monte Carlo light scattering simulator [1]. We use the Lorenz–Mie theory to generate the scattering, absorption, and the anistropy function of the microsphere solution used in the experiment. To match the experimental setup(see *parallelized speckle detection setup* in the Method section), we put detectors 9mm away from the illumination. The trajectory of the detected photon are recorded to study the volume region most detected photon has travelled through. Figure S1 (A) plots the center slice of the photon path that detected by two detectors placed 9mm away from the illumination. Although 12 detectors are used in the real setup, a cross section of photon trajectory from two detectors are presented here for a better visual illustration propose. Visualizations of 3D trajectories of detected photon from all 12 fibers, and 6,4, and 3 fibers are plotted in fig.S2(B). As expected, the light travel through banana-shaped paths, with the most sensitive region penetrates around 5mm deep. The surface fluence is plotted in fig. S1 (B), and a line-plot of the center line is provided. 10 billion photon is pumped into the surface center of the tissue phantom. The photon number is re-scaled to the 200mW 670nm illumination used in the experiment via the Planck–Einstein relation to give quantitative predictions of the photon number per speckle area per µs exposure on the tissue phantom surface. On average 9.4 photon per speckle per µs exiting the tissue phantom surface 9mm away from the illumination. The emperically measured number of photon using the SPAD array within this exposure time is less than 2 photon per pixel per µs, which is lower. This is due to the fiber detection and transmission efficiency, and the quantum efficiency of the SPAD. Hence, the measured photon number falls into the expected range. Figure S1 (C) gives the probabilistic distribution of the number of times photon gets scattered before detection, with an average number of scattering above 400 times. The distribution has a long tail, and no photon scattering less than 80 scattering are detected at 9mm source-detector seperation. Therefore, the simulation predicts all the detected light are highly scattered. However, in reality, as we used glass material to make both the cuvette and the probe surface, capturing leaking photon from the phantom

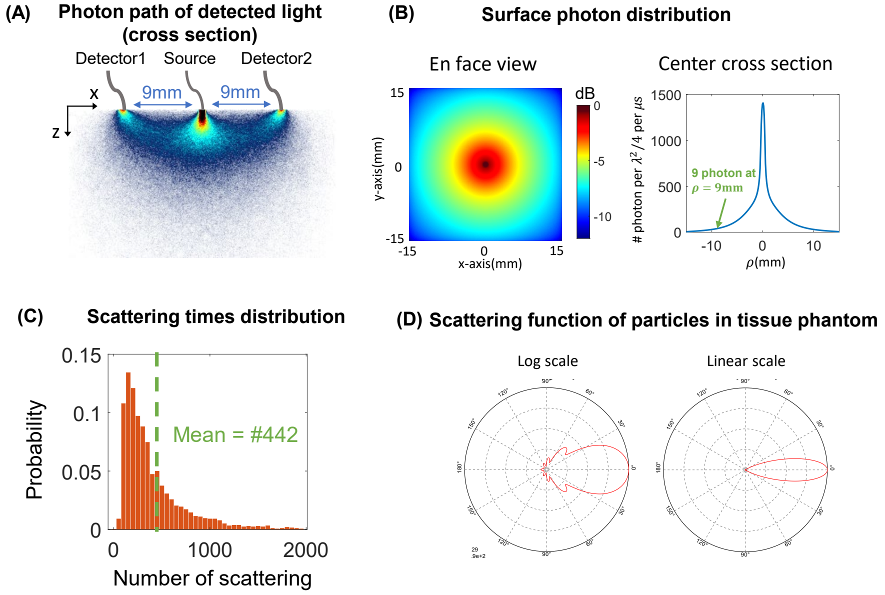

Figure S1: Monte carlo simulation of (A) Photon sensitive path of two source-detector pairs. (B) Left: En face view of the surface photon number distribution. Right: a center slice of the surface photon distribution. The y-axis is scaled to the number of backscattered photon on the surface per speckle area per micro-second when 200mW light is used. (C) The distribution of the number of scattering of detected photon. (D) The scattering function of the microsphere solution we used.

surface is also anticipated, as discussed in *Discussion* section in the main text. Figure S1 (D) shows the phase function for the microsphere solution calculated by the Mie scattering theory. In addition, we provide 3D trajectory of the detected photon and plot the imaging sensitivity of the PDCI system using different number of fiber detectors in fig.S2. These visualizations greatly help understand the imaging space of the system with different number of fiber detectors, and explains why employing more detectors can noticeably improve the imaging quality, as shown in the *Experimental validation* section in the main text.

S2 A model-based reconstruction

We compare our learning-based method with a model-based method. We assume the perturbation (object present subtract object absent) $\mathbf{b} \in \mathbb{R}^m$ generated by the DMD patterns is linearly related to the displayed pattern pixels $\mathbf{x} \in \mathbb{R}^n$ by $\mathbf{b} = \mathbf{W}\mathbf{x}, \mathbf{W} \in \mathbb{R}^{m\times n}$, where each column of $\mathbf{W} = [\mathbf{w}_1, \mathbf{w}_2, ..]$ are the perturbations generated by the decorrelating point source located at pixels $[x_1; x_2, ..]$ of $\mathbf{x}$; i.e., the perturbation generated by displaying both pattern 1 and pattern 2 is equal to the sum of the perturbations generated by displaying pattern 1 and 2 individually. While analytical Green's functions of diffuse correlation equation(DCE) exist for simple media geometry, such as infinite or semi-infinite geometries, it is *not* available for most arbitrary tissue shapes. Moreover, as mentioned in the text, the diffuse correlation equation is not a good approximation

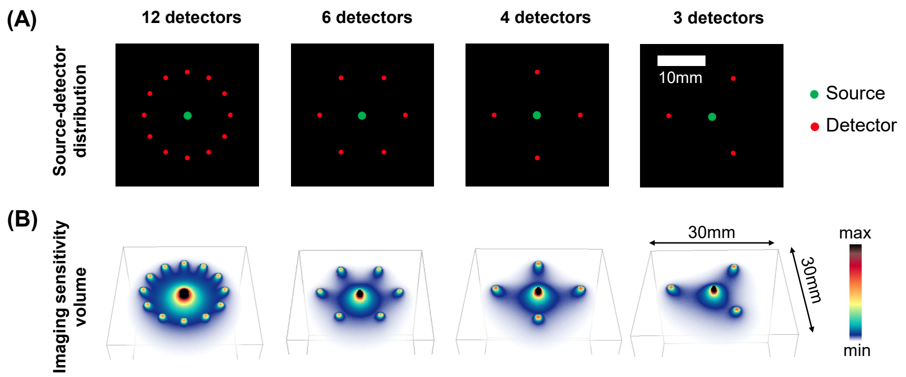

Figure S2: (A) PDCI system with different number of fiber detectors. The source-detector configurations are used to generate Fig.8 in the main article. (B) shows the imaging space of the PDCI systems with different number of detectors, with 12-fiber covers the most volume underneath. Images in each row share the same scale bar.

of the transported correlation equation for small source-detector separations used here. Hence, we measure the perturbation generated from each positions over a 0.67mm-pitch grid by turning on a small 1.36mm-radius circular DMD area centered at each grid point in sequence, which is smaller than the expected achievable resolution [2]). We apply ℓ_1 and isotropic total variation penalties to regularize the ill-posed reconstruction. Such regularizations has been successfully applied to diffuse optical tomography to improve reconstruction quality [3, 4]. The inverse problem can be formulated as

$$\mathbf{x} = \arg\min_{\mathbf{x}} \frac{1}{2}\|\mathbf{W}\mathbf{x} - \mathbf{b}\|_2^2 + \beta\|\mathbf{x}\|_1 + \gamma\|\mathbf{x}\|_{\text{tv}}. \tag{1}$$

To solve this, we use a variable splitting method. We first rewrite the problem as

$$\mathbf{x}, \mathbf{y}, \mathbf{z} = \arg\min_{\mathbf{x},\mathbf{y},\mathbf{z}} \frac{1}{2}\|\mathbf{W}\mathbf{x} - \mathbf{b}\|_2^2 + \beta\|\mathbf{y}\|_1 + \gamma\|\mathbf{z}\|_{\text{tv}}, \quad \text{s.t. } \mathbf{x} = \mathbf{y}, \mathbf{x} = \mathbf{z}, \tag{2}$$

which is equivalent to solving the augmented Lagrangian

$$\mathbf{x}, \mathbf{y}, \mathbf{z} = \arg\min_{\mathbf{x},\mathbf{y},\mathbf{z};\mathbf{u},\mathbf{v}} \mathcal{L}(\mathbf{x}, \mathbf{y}, \mathbf{z}; \mathbf{u}, \mathbf{v}), \tag{3}$$

where

$$\begin{aligned} \mathcal{L}(\mathbf{x}, \mathbf{y}, \mathbf{z}; \mathbf{u}, \mathbf{v}) = {} & \frac{1}{2}\|\mathbf{W}\mathbf{x} - \mathbf{b}\|_2^2 + \beta\|\mathbf{y}\|_1 + \gamma\|\mathbf{z}\|_{\text{tv}} \\ & + \mathbf{u}^\top(\mathbf{x} - \mathbf{y}) + \mathbf{v}^\top(\mathbf{x} - \mathbf{z}) + \frac{\rho_1}{2}\|\mathbf{x} - \mathbf{y}\|_2^2 + \frac{\rho_2}{2}\|\mathbf{x} - \mathbf{z}\|_2^2. \end{aligned} \tag{4}$$

This can be solved efficiently using the alternating direction method of multipliers(ADMM) [5] encapsulated in algorithm 1, where the primal variables minimization steps can be simplified as

$$\mathbf{x} = \arg\min_{\mathbf{x}} \frac{1}{2}\|\mathbf{W}\mathbf{x} - \mathbf{b}\|_2^2 + \frac{\rho_1}{2}\|\mathbf{x} - \mathbf{y} + \mathbf{u}\|_2^2 + \frac{\rho_2}{2}\|\mathbf{x} - \mathbf{z} + \mathbf{v}\|_2^2, \tag{5}$$

$$\mathbf{y} = \arg\min_{\mathbf{y}} \beta\|\mathbf{y}\|_1 + \frac{\rho_1}{2}\|\mathbf{x} - \mathbf{y} + \mathbf{u}\|_2^2, \tag{6}$$

$$\mathbf{z} = \arg\min_{\mathbf{z}} \beta\|\mathbf{z}\|_{\text{tv}} + \frac{\rho_2}{2}\|\mathbf{x} - \mathbf{z} + \mathbf{v}\|_2^2, \tag{7}$$

respectively. Equation 5 has a close-form solution

$$\mathbf{x} = (\mathbf{W}^{\mathrm{T}}\mathbf{W} + \rho_1\mathbf{I} + \rho_2\mathbf{I})^{-1}(\rho_1(\mathbf{y} - \mathbf{u}) + \rho_2(\mathbf{z} - \mathbf{v}) + \mathbf{W}^{\mathrm{T}}\mathbf{b}). \tag{8}$$

Equation 6 also has a close-form solution

$$\mathbf{y} = \mathcal{S}(\mathbf{y}, {}^{2\beta}/{}_{\rho_1}), \tag{9}$$

where $\mathcal{S}(\cdot, \lambda)$ is the soft-threshold function with a threshold λ. Unfortunately the proximal of the TV regularization in equation 7 does not have a close-form solution; however, we can solve it efficiently using the method proposed by Beck and Teboulle [6] that converges in 10 iterations.

S3 Liquid phantom optical and dynamic property

Here we present a way to estimate the scattering, absorption, and decorrelating properties of the polystyrene microsphere liquid phantom we use in the experiments. Our phantom is made of 1-micronmeter polystyrene microspheres suspension with a concentration of $4.55 \times 10^6\#/\text{mm}^3$. Using one of the most popular reported complex refractive index of polystyrene (1.584-0.0004i) measured by Ma *et.al.* [7], the scattering(μ_s') and absorption coefficient(μ_a) of the polystyrene microsphere solution can be calculated with the Lorenz-Mie theory [8], which results in an calculated $\mu_s' = 0.7\text{mm}^{-1}$ and $\mu_a = 0.02\text{mm}^{-1}$. However, as the extinction coefficient of the polystyrene in 670nm wavelength is very small, a tiny variance (on the order of 10×-4) caused by manufacturing process inconsistency or discrepancy can result in noticeable difference in the absorption coefficient. Hence, we experimentally measure the absorption coefficient using a relation between surface diffuse reflectance and source-detector distance derived from the diffusion equation [9]

$$\ln(\rho^2 I_\rho) = -\mu_{\mathit{eff}} + I_0, \tag{10}$$

Algorithm 1 Proposed ADMM-based reconstruction method

Input: initial guess $\mathbf{x}^0$, system matrix $\mathbf{W}$, measurement $\mathbf{b}$, number if iteration T .
Init: $\mathbf{y}^0 = \mathbf{x}^0, \mathbf{z}^0 = \mathbf{x}^0$, $\mathbf{u}^0 = \mathbf{0}$, $\mathbf{v}^0 = \mathbf{0}$.
for $t = 1, 2, \ldots, T$ **do**
    $\mathbf{x}^t = \arg\min_{\mathbf{x}} \mathcal{L}(\mathbf{x}^{t-1}, \mathbf{y}^{t-1}, \mathbf{z}^{t-1}; \mathbf{u}^{t-1}, \mathbf{v}^{t-1})$ ▷ Eq.8
    $\mathbf{y}^t = \arg\min_{\mathbf{y}} \mathcal{L}(\mathbf{x}^t, \mathbf{y}^{t-1}, \mathbf{z}^{t-1}; \mathbf{u}^{t-1}, \mathbf{v}^{t-1})$ ▷ Eq.9
    $\mathbf{z}^t = \arg\min_{\mathbf{z}} \mathcal{L}(\mathbf{x}^t, \mathbf{y}^t, \mathbf{z}^{t-1}; \mathbf{u}^{t-1}, \mathbf{v}^{t-1})$
    $\mathbf{u}^t = \mathbf{u}^{t-1} + \mathbf{x}^t - \mathbf{y}^t$ ▷ Dual ascent
    $\mathbf{v}^t = \mathbf{v}^{t-1} + \mathbf{x}^t - \mathbf{z}^t$ ▷ Dual ascent
end for
Output: $\mathbf{x}^T$

where ρ is the source-detector distance. $\mu_{eff} = \sqrt{3\mu_s'\mu_a}$ is the effective attenuation coefficient. I_ρ and I_0 are the surface diffuse reflectance at ρ and 0, respectively. ln is the nature logarithmic function. Fig. S3 plots the experimentally measured ln $(\rho^2 I_\rho)$ as a function of the source-detector separation. Fitting the points with a straight line, we can derive the absorption coefficent to be $\mu_a = 0.01\text{cm}^{-1}$.

Next, we want to estimate the dynamic property of the media. Since we use a 0.9cm source-detector separation in the experiment, a Monte Carlo method is used to give more accurate result [10]. Consider a photon n experience its ith scattering inside the medium m, resulting a momentum transfer $\mathbf{q}^i_{n,m}$ and a traveling path length $l^i_{n,m}$, where $\mathbf{q} = \mathbf{k}_{out} - \mathbf{k}_{in}$ with $\mathbf{k}_{out}$ and $\mathbf{k}_{in}$ are wave-vectors scattered from and towards the colli- sion, respectively. The total dimensionless *momentum transfer* an photon *traveling path* d

length of photon n inside medium m can be written as $Y_{n,m} = \sum_{i=1} (q^i_{n,m})^2/(2k_m^2)$ and $L_{n,m} = \sum_{i=1} l^i_{n,m}$, respectively, with each individual $q^i_{n,m}$ and $l^i_{n,m}$ tracked from the Monte Carlo simulation. Therefore, the field correlation can be calculated as [10]

$$G_1(\tau) = \frac{1}{N_p}\sum_{n=1}^{N_p} \exp\Big(-\frac{1}{3}\sum_{m=1}^{M} Y_{n,m}k_m^2\langle\Delta r_m^2(\tau)\rangle\Big)\exp\Big(\sum_{m=1}^{M} -\mu_{a_m}L_{n,m}\Big), \tag{11}$$

where M is the number of different tissue types, and N_p is the number of detected photons. k_m and μ_{a_m} are the wave-number and absorption coefficient in medium m. Since we are estimating the property for the background media, which is homogeneous, $M = 1$ in this case. Further, we assume the polystyrene bead suspension experience Brownian motion,

which makes $\langle\Delta r_m^2(\tau)\rangle = 6D_v\tau$. From field correlation curves, we can compute the normalized intensity correlation using the Siegert relation [11]

$$g_2(\tau) = 1 + |g_1(\tau)|^2, \tag{12}$$

where $g_1(\tau) = G_1(\tau)/G_1(0)$ is the normalized field correlation. Fitting the experimentally measured $g_2(\tau)$ with simulated ones, we derive the diffusion coefficient for the media $D_v = 1.5 \times 10^{-6}\text{mm}^2/\text{s}$, which is close to the diffusion coefficient in small animals [12].

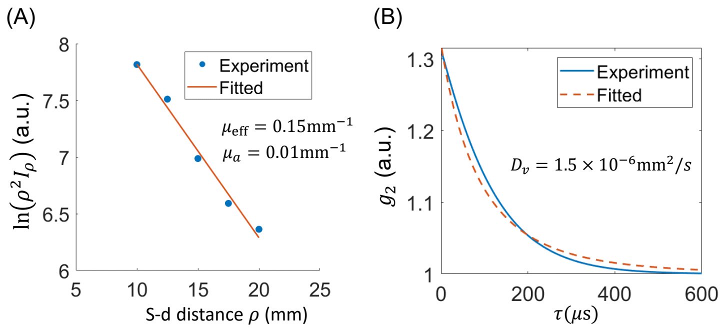

Figure S3: Validation of diffusion model and intensity autocorrelation function (A) Experimentally measured ln $(\rho^2 I_\rho)$ as a function of source-detector seperation. Fitting measured points gives an estimated $\mu_a = 0.01\text{cm}^{-1}$. (B) Fitting intensity autocorrelation $g_2(\tau)$ using simulation gives a predicted Brownian diffusion coefficient $D_v = 1.5 \times 10^{-6}\text{mm}^2/\text{s}$.

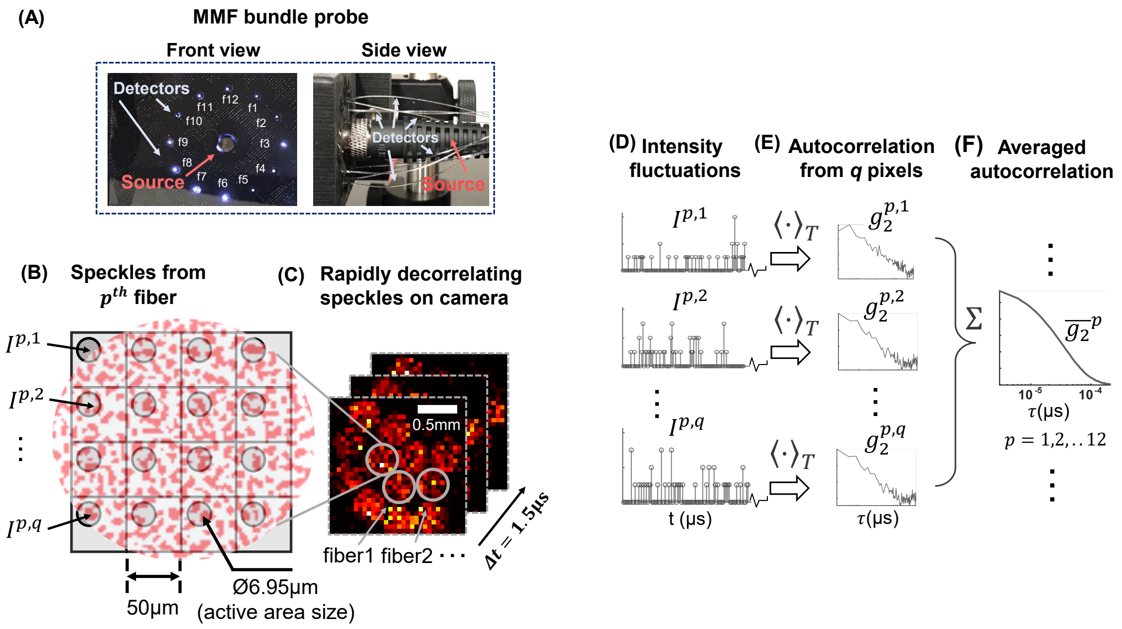

Figure S4: Data preprocessing flow for our parallelized speckle detection system. (A) Photos of fiber bundle probe, showing 12 detectors radially positioned around light delivery fiber in center. Light collected from each positions in (A) are mapped to and collected by the SPAD array, as displayed in (C). (B) shows a few frames of the raw data captured by the 32×32 SPAD array camera at a $1.5\mu s$ exposure. (C) illustrates the SPAD pixels that records the speckle fluctuations from the detector fiber p. (D) some representative time-resolved photon counting measurements from each SPAD pixel. The normalized intensity temperal autocorrelation curve for each pixel is calculated using the eq.**??** as plotted in (E). All the computed correlations from SPAD pixels that measures the speckle p are averaged to generate a relatively smooth autocorrelation $\overline{g_2}^p$ for the surface location $p = 1, 2, .., 12$.

References

[1] Joakim Jönsson and Edouard Berrocal, "Multi-scattering software: part i: online accelerated monte carlo simulation of light transport through scattering media," *Optics Express*, vol. 28, no. 25, pp. 37612–37638, 2020.

[2] Wenhui Liu, Ruobing Qian, Shiqi Xu, Pavan Chandra Konda, Joakim Jönsson, Mark Harfouche, Dawid Borycki, Colin Cooke, Edouard Berrocal, Qionghai Dai, et al., "Fast and sensitive diffuse correlation spectroscopy with highly parallelized single photon detection," *APL Photonics*, vol. 6, no. 2, pp. 026106, 2021.

[3] Teresa Correia, Juan Aguirre, Alejandro Sisniega, Judit Chamorro-Servent, Juan Abascal, Juan J Vaquero, Manuel Desco, Ville Kolehmainen, and Simon Arridge, "Split operator method for fluorescence diffuse optical tomography using anisotropic diffusion regularisation with prior anatomical information," *Biomedical optics express*, vol. 2, no. 9, pp. 2632–2648, 2011.

[4] Shiqi Xu, KM Shihab Uddin, and Quing Zhu, "Improving dot reconstruction with a born iterative method and us-guided sparse regularization," *Biomedical optics express*, vol. 10, no. 5, pp. 2528–2541, 2019.

[5] Stephen Boyd, Neal Parikh, and Eric Chu, *Distributed optimization and statistical learning via the alternating direction method of multipliers*, Now Publishers Inc, 2011.

[6] Amir Beck and Marc Teboulle, "A fast iterative shrinkage-thresholding algorithm for linear inverse problems," *SIAM journal on imaging sciences*, vol. 2, no. 1, pp. 183–202, 2009.

[7] Xiaoyan Ma, Jun Q Lu, R Scott Brock, Kenneth M Jacobs, Ping Yang, and Xin-Hua Hu, "Determination of complex refractive index of polystyrene microspheres from 370 to 1610 nm," *Physics in medicine & biology*, vol. 48, no. 24, pp. 4165, 2003.

148 [8] Hendrik Christoffel Hulst and Hendrik C van de Hulst, *Light scattering by small particles*, Courier Corporation, 1981.

[9] Davide Tamborini, Parisa Farzam, Bernhard B Zimmermann, Kuan-Cheng Wu, David A Boas, and Maria Angela Franceschini, "Development and characterization of a multidistance and multiwavelength diffuse correlation spectroscopy system," *Neurophotonics*, vol. 5, no. 1, pp. 011015, 2017.

[10] David A Boas, Sava Sakadžić, Juliette J Selb, Parisa Farzam, Maria Angela Franceschini, and Stefan A Carp, "Establishing the diffuse correlation spectroscopy signal relationship with blood flow," *Neurophotonics*, vol. 3, no. 3, pp. 031412, 2016.

[11] P-A Lemieux and DJ Durian, "Investigating non-gaussian scattering processes by using nth-order intensity correlation functions," *JOSA A*, vol. 16, no. 7, pp. 1651–1664, 1999.

[12] Turgut Durduran, Regine Choe, Wesley B Baker, and Arjun G Yodh, "Diffuse optics for tissue monitoring and tomography," *Reports on Progress in Physics*, vol. 73, no. 7, pp. 076701, 2010.
